# Effects of cigarette smoke and HIV-1 factors on blood-brain barrier integrity and permeability in an in vitro model

**DOI:** 10.1007/s13365-025-01295-2

**Published:** 2026-01-02

**Authors:** Joseph D. Walker, Binu Tharakan, Talib Saafir, Walter Royal

**Affiliations:** 1 Department of Neurobiology, Morehouse School of Medicine, Atlanta, GA USA; 2 Neuroscience Institute, Morehouse School of Medicine, Atlanta, GA USA; 3 Department of Surgery, Morehouse School of Medicine, Atlanta, GA USA; 4 Department of Biology and Center for Brain Health Research, Morgan State University, Baltimore, MD USA

**Keywords:** Blood-brain barrier, HIV, Transgenic rat, Cigarette smoke

## Abstract

Background: HIV-associated neurocognitive impairment (HAND) is a common complication of HIV-1 infection, which can be exacerbated by exposure to cigarette smoke (CS). Tight junction proteins (TJPs) of the blood-brain barrier (BBB) play a crucial role in maintaining BBB integrity and preventing the entry of circulating toxic factors, including those resulting from HIV-1 infection, into the central nervous system. Both CS exposure and HIV-1 infection can independently disrupt TJPs and compromise BBB integrity; however, the combined or individual effects of these factors on BBB TJPs remain poorly understood. Methods: An in vitro BBB comprised of Sprague-Dawley rat brain microvascular endothelial cell (RBMVEC) transwell cultures was exposed to wild-type (WT) and HIV-1 transgenic (TG) rat sera, alone or in combination with cigarette smoke extract (CSE) and analyzed for trans-endothelial electrical resistance (TEER) and paracellular permeability to 10 kDa fluorescein isothiocyanate (FITC)-dextran. Immunofluorescence staining was performed to assess the effects of treatment on the cellular localization and expression of the TJPs, “zonula occludens-1 (ZO-1) and claudin-5. Results: Pretreatment TEER measures were significantly higher for cultures treated with WT serum alone compared to those treated with TG serum or with CSE. Compared to pretreatment, TEER measures were significantly reduced by treatment with WT serum alone, CSE alone, WT serum + CSE, and TG serum + CSE. TG serum alone or TG serum + CSE resulted in statistically significant increased permeability compared to WT serum. All treatments decreased TJP staining intensity, and, in some cases, altered TJP localization. These effects were most prominent following incubation with either CSE alone, TG serum alone, or TG serum + CSE. Conclusions: CSE and TG serum induced separate and additive toxic effects on BBB function and integrity, which may underlie mechanisms that are associated with more severe HAND among HIV+ cigarette smokers.

## Introduction

According to the Centers for Disease Control and Prevention (CDC), the prevalence of current cigarette smoking in the United States has decreased from 20.9% in 2005 to 11.5% in 2021. However, this still represents an estimated 28.3 million U.S. adults, with nearly 12 out of every 100 individuals aged 18 or older being habitual cigarette smokers (VanFrank et al. 2024). Additionally, more than 16 million of these individuals currently live with a smoking-related disease. Notably, the frequency of cigarette smokers among the HIV-1 infected population remains 2–3 times higher than the national average (Rahmanian et al. 2011). Studies have revealed that those with HIV infection are 2–3 times more likely to be habitual smokers, amplifying the already considerable burden of their condition. 

The development and widespread use of effective antiretroviral therapy (ART) has led to dramatic decreases in the frequency and severity of HIV-related complications and lower mortality. In particular, the incidence and prevalence of one of the most devastating HIV-related complication, HIV-associated dementia, has decreased. In recent decades, it has been recognized that HIV-associated cognitive, motor, and behavioral dysfunction—along with abnormalities detected through objective testing—can be used to classify affected individuals into one of three categories of increasing severity: asymptomatic neurocognitive impairment, minor neurocognitive disorder, or HIV-associated dementia. This clinical spectrum has been termed HIV-associated neurocognitive disorder (HAND) (Antinori et al. 2007), which, due to the efficacy of ART, results in its occurrence in nearly half of PLHIV and the majority of individuals having the milder forms (Heaton et al. 2010). Moreover, recent studies have demonstrated that HAND is exacerbated by cigarette smoking (Chang et al. 2017; Harrison et al. 2017). This can occur as a result of suboptimal treatment adherence, reduced treatment efficacy, increased toxicity, and accelerated aging in PLHIV who smoke (Rahmanian et al. 2011). Neurotoxicity in HIV infection arises from the release of cytokines and other inflammatory and oxidative stress mediators by infected and activated immune and glial cells, as well as from the direct toxic effects of HIV proteins (Anderson et al. 2002). Similarly, cigarette smoking promotes the development of inflammation and reactive oxygen species that can enter the brain and induce toxicity (Barbieri et al. 2011; Petrescu et al. 2010). Notably, the effects of nicotine—an addictive compound commonly found in cigarettes—may depend on the timing of exposure, further influencing the extent and nature of neurotoxicity. (Cao et al. 2024; Zhang et al. 2022). In addition, chronic nicotine exposure in the nervous system has been linked to dysregulated nicotinic receptor expression and function, which can override nicotine’s expected anti-inflammatory and neuroprotective effects—ultimately promoting proinflammatory responses and neuronal damage. (Ballester et al. 2012; Bracci et al. 1992; Capo-Velez et al. 2018; Marks et al. 1993; Rios et al. 2021).

Under normal circumstances, the brain is protected from exposure to toxic factors and activated immune cell entry by the blood-brain barrier (BBB), which is comprised of non-fenestrated endothelial cells, pericytes, and astrocytes. The endothelial cells are connected by tight junction complexes which play a crucial role in maintaining the integrity and selective permeability of the BBB, thereby regulating the exchange of molecules and cells between the blood and the brain. Tight junction proteins create rows of overlapping occlusions between adjacent endothelial cells, forming a physical barrier that prevents paracellular diffusion of polar molecules and macromolecules into the central nervous system. The key tight junction proteins expressed at the BBB include the claudins, occludin, and junctional adhesion molecules (JAMs), which interact with each other as well as with peripheral proteins, such as zonula occludin (ZO)-1, -2, and − 3 (Fanning et al. 1998). Among the claudin family, claudin-5 is considered the dominant tight junction protein at the BBB, with minor contributions from claudin-3 and − 12 (Greene et al. 2019). Occludin, another transmembrane protein, regulates the permeability and charge selectivity of the BBB. JAMs are involved in leukocyte transmigration across the BBB and in maintaining endothelial cell polarity. The cytoplasmic proteins ZO-1, -2, and − 3 link the transmembrane proteins to the actin cytoskeleton, modulating their expression, localization, and function. Tight junction proteins are dynamic structures that can be modulated by various physiological and pathological stimuli, such as cytokines, growth factors, oxidative stress, hypoxia, infection, and inflammation.

In studies performed in an HIV-1 transgenic (TG) rat model of HIV-1 infection established in Fischer rats (Reid et al. 2001), it was found that chronic CS exposure resulted in increased plasma levels of IL-1β and IL-6 as compared to wild-type control rats (Royal et al. 2018). Notably, similar effects were observed in rats chronically exposed to subcutaneous nicotine, highlighting nicotine’s independent impact. Together, nicotine, CS, and HIV infection have all been shown to exert profound effects on the integrity and function of the blood-brain barrier (BBB).

The overall objective of this study is to enhance the understanding of how cigarette smoke (CS) affects blood BBB function in the context of HIV-1 infection. For this study a well-established in vitro BBB model employing rat brain microvascular endothelial cells (RBMVECs) was utilized to analyze the effects of HIV-1 infection and CS on BBB structure and function. The RBMVECs cultures were incubated with serum from either TG or wild-type (WT) rats alone or in the presence of cigarette smoke extract (CSE). These studies revealed effects in this model that provide insights into potential molecular mechanisms underlying BBB disruption due to exposure CS and HIV-1.

## Results

### RBMVEC cultures

**Fig. 1:**
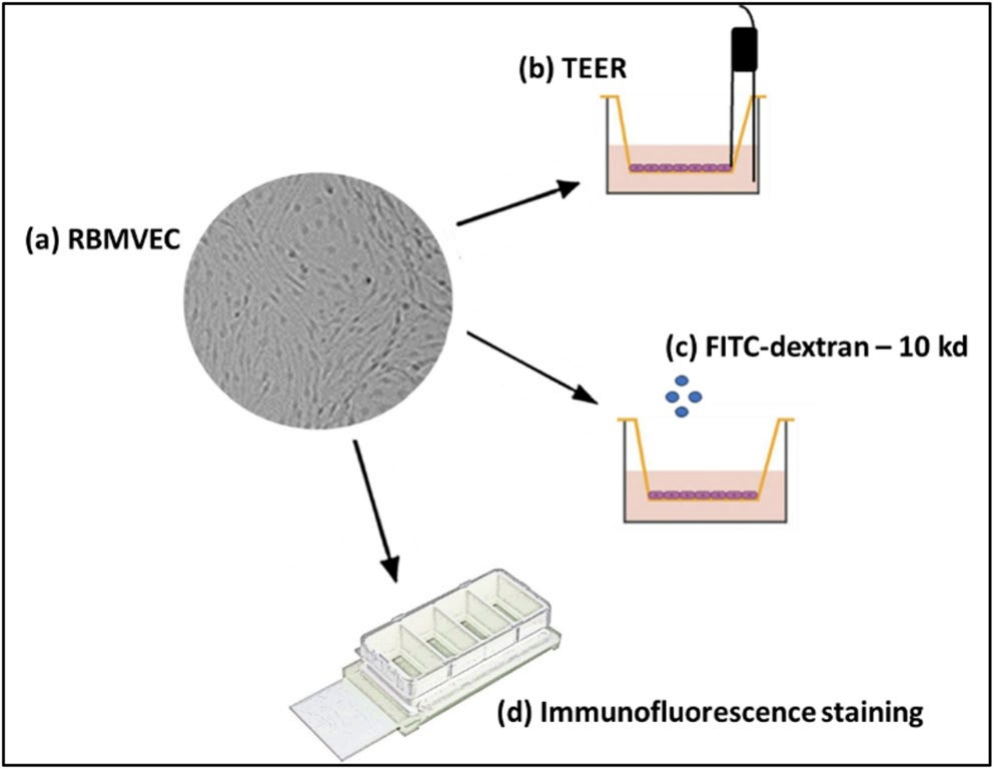
Depiction of the sequence of experiments performed. (**a**) Phase contrast image of confluent Sprague-Dawley rat brain microvascular endothelial cells were seeded on (**b**) transwell inserts for measurement of Trans-Epithelial/Endothelial Electrical Resistance (TEER). The cells were also cultured on (**c**) chambered cell chambered cell culture slides, which were subsequently processed for immunofluorescence imaging following staining with specific primary antibodies against ZO-1 and claudin-5. See main text for details. Modified from Biorender

Figure 1 shows a flow chart outlining the experimental approach. Passage 3 Sprague Dawley RBMVEC (shown in the representative phase contrast image, Fig. 1a), were utilized to seed replicate chamber slides and transwell inserts for immunostaining and TEER analyses.

### Effects of TG serum and CSE on RBMVEC TEER measures

**Fig. 2:**
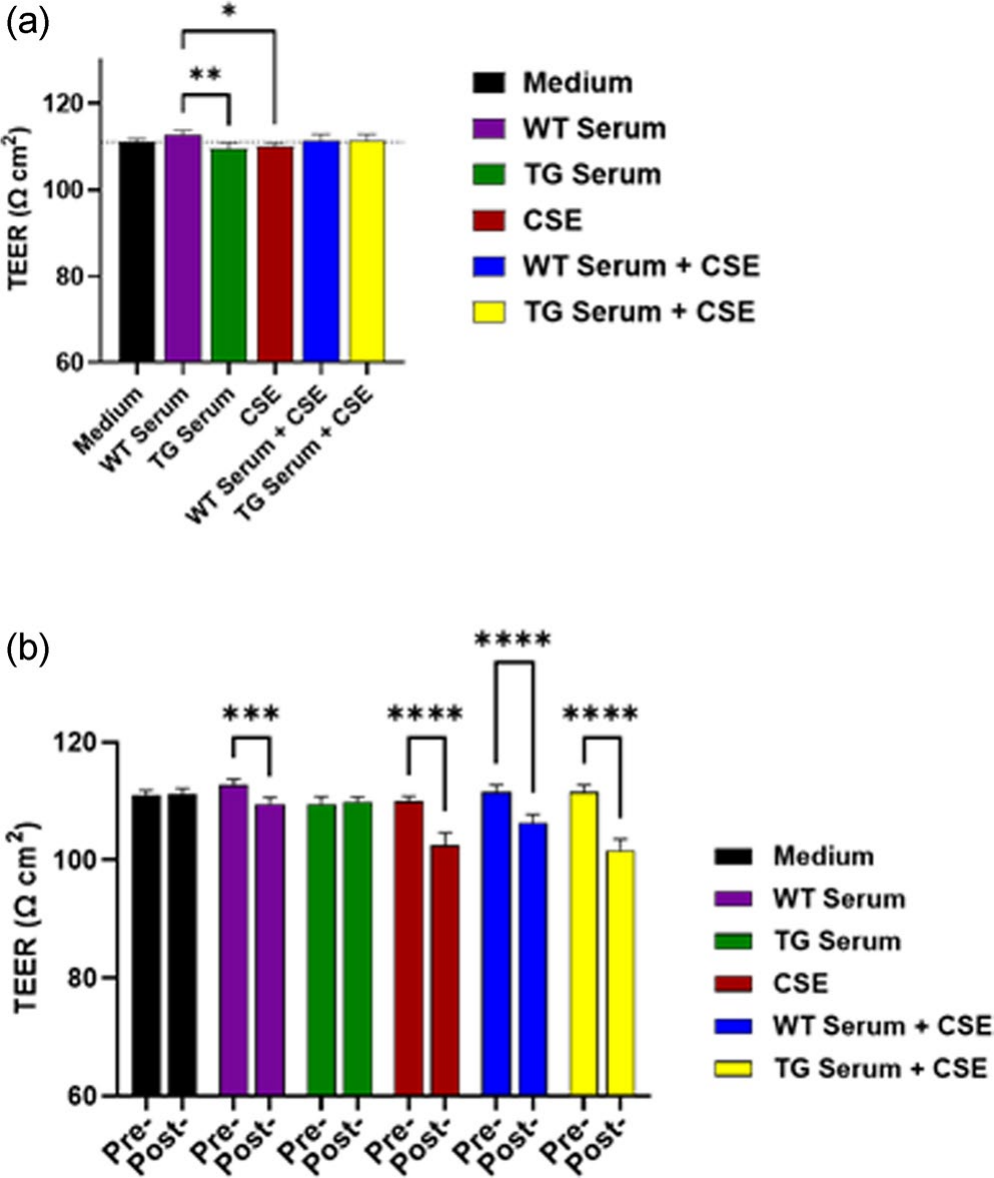
(**a**) Comparison of the pretreatment TEER measures for cultures subsequently incubated with either medium alone, WT serum, TG serum, CSE, WT serum + CSE, or TG serum + CSE. (**b**) Comparisons of the pretreatment versus posttreatment TEER (Ω cm2) value for each group. Posttreatment TEER values were lower for cells treated with WT serum, CSE, WT serum + CSE, and TG serum + CSE as compare to pretreatment. * = *p* < 0.05; ** = *p* < 0.01; *** = *p* < 0.001; **** = *p* < 0001

Studies were performed to determine the differential effects of either TG rat serum and CSE on TEER in RBMVEC. Pretreatment TEER measures were significantly higher for cultures treated with WT serum alone than those treated with TG serum or with CSE (Fig. 2a). Figure 2b shows the comparisons of the pretreatment versus posttreatment TEER measures. This analysis showed TEER measurements that, versus the matched pretreatment control, were significantly lower for cultures treated with WT serum alone, CSE alone, WT serum + CSE, and TG serum + CSE.

### Effects of serum and CSE exposures on permeability in the BBB model

**Fig. 3:**
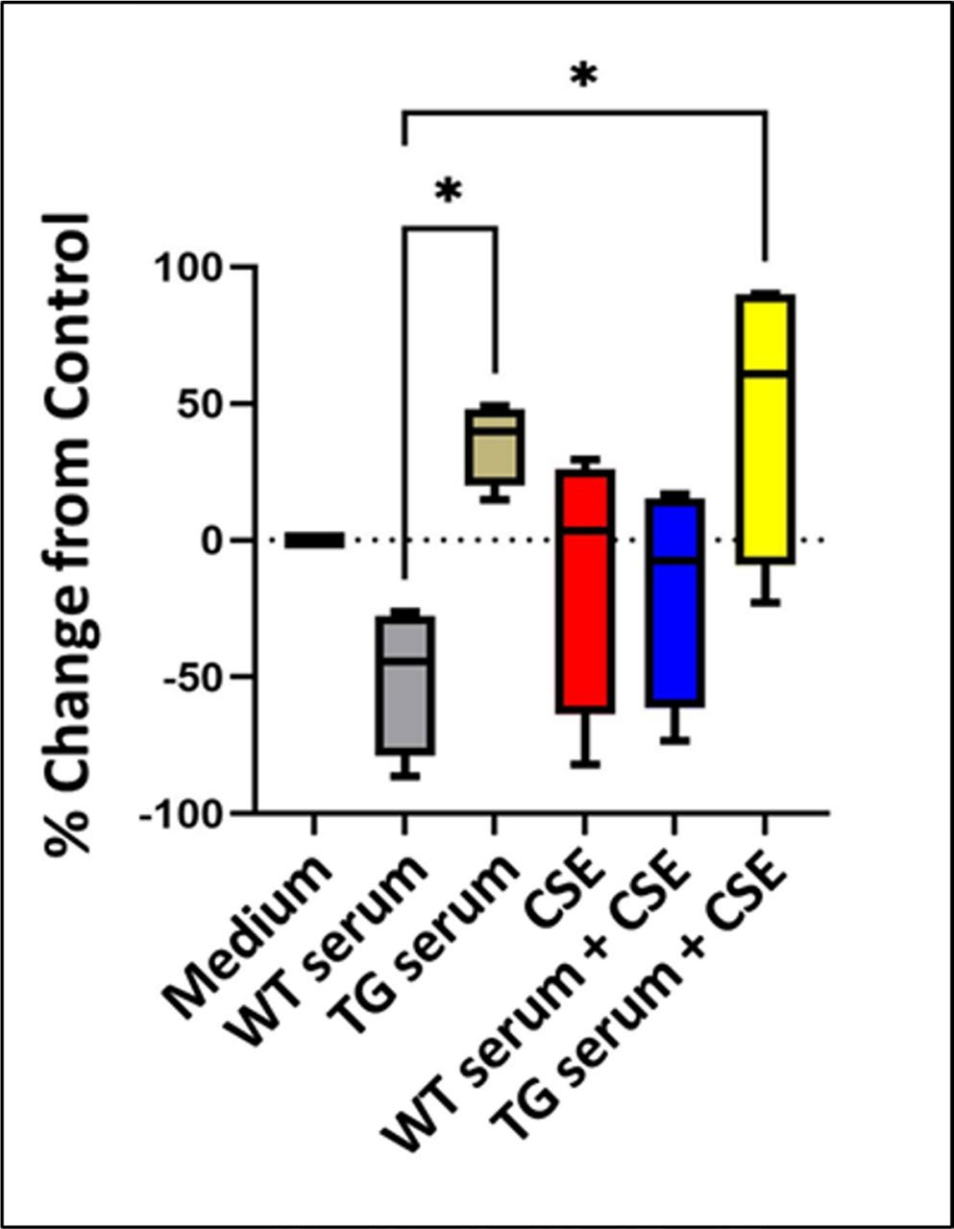
Analysis of the permeability of the transwell cultures to FITC dextran. There was a non-significant decrease in permeability after incubation with WT rat serum alone as compared to the negative control group. The permeability for cultures incubated with either HIV TG rat serum alone or HIV-1 TG rat serum incubated with CSE was significantly greater than for cells incubated with WT serum. * = *p* < 0.05

To assess the integrity of the endothelial cell monolayer, the control and treated cultures were analyzed for permeability to ~ 10 kDa FITC-dextran (Fig. 3). As previously described in the Methods section, the FITC dextran was placed in the apical side of the transwell after the 24-hour treatments. After allowing the cultures to incubate, permeability to the FITC-dextran was calculated as the percent change in the fluorescence intensity measured on media collected on the basolateral side for the treated versus the control group. Cells exposed to WT serum showed an approximately 50% decrease in permeability as compared to the negative control (incubation with medium alone); however, this difference was not statistically significant. Incubation with either TG serum alone or TG serum + CSE resulted in statistically significant increases in permeability compared to WT serum (*p* = 0.0372 and *p* = 0.0152, respectively). There were no statistically significant differences in permeability measures for any of the treatment groups versus the negative control group and no difference in measures for any other treatment and control group comparisons.

### WT and TG rat serum and CSE effects on ZO-1 localization and expression

**Fig. 4:**
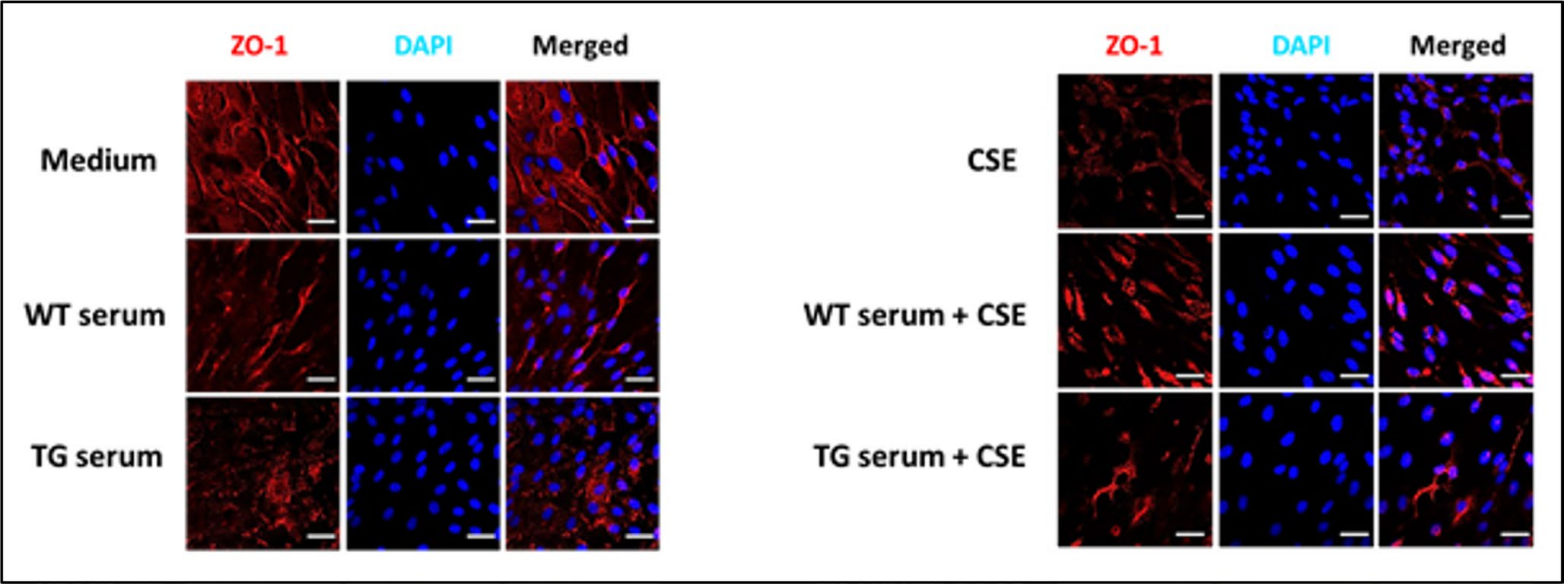
Immunofluorescence staining of post-treatment chamber slide cultures for ZO-1. Representative fluorescence microscopic images of RBMVEC cultures incubated with (left) either medium, WT serum, TG serum or (right) with either CSE, WT rat serum + CSE or TG serum + CSE. The cells were fixed in paraformaldehyde and then incubated with ZO-1- specific primary and Alexa Fluor 568-conjugated secondary antibodies and stained with DAPI, (20x magnification with additional 1.5-fold zoom; scale 20 μm). See the main text for the detailed methods and results. (Error bars = mean + standard deviation)

In normal confluent cells, ZO-1 localizes to regions of cell contact where it promotes the formation of tight junctions (Tornavaca et al. 2015). To determine the effects of exposing RBMVEC to WT or TG rat serum with or without CSE or to CSE alone, the control and treated cells were fixed with 4% paraformaldehyde and then stained for immunofluorescence imaging using primary antibodies against ZO-1, with nuclei stained with DAPI (Fig. 4). Cells treated with medium alone showed ZO-1 staining of cell plasma membrane in associated with diffuse cytoplasmic staining and primarily eccentrically located nuclei. In some cases, there was also perinuclear staining that was continuous with the plasma membrane as well as infrequent intranuclear staining. Treatment with WT serum was associated with a loss of staining at intercellular contact regions, and instead, shorter stretches of linear staining were observed, which were also continuous with staining in the perinuclear regions. Staining following exposure to TG rat serum resulted in the presence of only punctate staining of perinuclear areas.

To examine the effects of BBB exposure to CS, cell cultures were incubated with CSE alone or in the presence of WT or TG rat serum. Incubation with CSE alone also resulted the absence of staining at intercellular contacts as well as with short stretches of linear staining and punctate staining of a few nuclei. Exposing the cells to WT serum plus CSE resulted in the loss of nearly all linear staining and the development of large and prominently stained nuclei. Cells treated with TG serum containing CSE showed markedly diminished staining overall with areas of linear staining and moderately enlarged nuclei, some which were stained for ZO-1. These findings suggest that there was an impact of CSE exposure on cell viability.

### Effects of WT and TG rat serum and CSE on Claudin-5 localization and expression

**Fig. 5:**
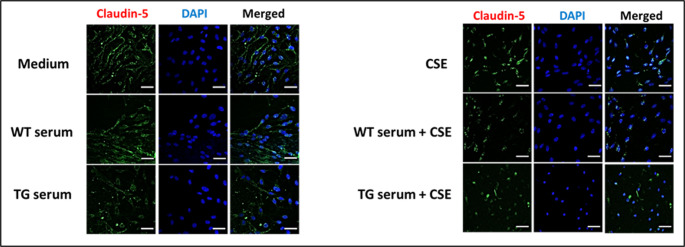
Immunofluorescence staining of post-treatment chamber slide cultures for claudin-5. Representative fluorescence microscopic images of RBMVEC cultures incubated with (left) either medium, WT serum, TG serum or (right) with either CSE, WT rat serum + CSE or TG serum + CSE. The cells were fixed in paraformaldehyde and then incubated with claudin-5-specific primary and Alexa Fluor 488-conjugated secondary antibodies and then stained with DAPI, (20x magnification with additional 1.5-fold zoom; scale 20 μm). See the main text for the detailed methods and results. (Error bars = mean + standard deviation)

Immunofluorescence staining was also performed for the tight junction protein claudin-5 (Fig. 5). For cells treated with medium alone, staining was observed at cell contact regions. These cells also had eccentric nuclei with perinuclear staining and punctate nuclear staining. Treatment with WT rat serum resulted in cells with decreased cytoplasmic staining and also with perinuclear and intranuclear staining. Cells that were treated with either WT rat serum + CSE, TG rat serum without CSE, or TG serum + CSE showed, respectively, lower levels of staining. Treatment with TG rat serum alone resulted in the least overall staining with areas of punctate staining distributed over non-nuclear areas, and cells treated with TG rat serum plus CSE contained overall smaller nuclei. These finding suggest that cell viability was likely diminished by exposure to CSE.

## Discussion

Neurological and neurocognitive dysfunction continue to be prevalent among individuals living with HIV-1 infection (Heaton et al. 2010), a condition often exacerbated by cigarette smoking (Chang et al. 2017; Harrison et al. 2017). Studies have revealed that those with HIV-1 are 2–3 times more likely to be habitual smokers (Rahmanian et al. 2011), amplifying the already considerable burden of their condition. In this study we analyzed the effects of CS exposure and HIV-1 infection on BBB function and structure using an in vitro BBB cell culture model with the cells exposed to TG rat serum and CSE. HIV-1 does not infect BBB endothelial cells in vivo, but HIV proteins can directly disrupt endothelial and mucosal barriers (Anand et al. 2018; Kanmogne et al. 2007). Therefore, analyzing the effects of circulating factors was an appropriate approach to pursue in this model. We found that cell injury may have resulted from exposure to CSE and that treatment with CSE alone or in the presence of WT or TG serum resulted in TEER measurements that were overall lower than for the matched control cultures. These findings suggest that soluble factors are present in TG rat serum and CSE that can induce significant toxicity to the endothelial cells and that combining these two factors resulted in toxic effects that were additive. Notably, exposure to WT sera alone resulted in TEER measures that were lower than for the matched pretreatment control. However, these measures were also significantly higher than the TG sera and CSE measures. This suggests that the pretreatment and posttreatment TEER difference for the WT serum group may have occurred either by chance or due to some other occurrence, such as an overgrowth of the cell monolayer. Had there been no difference in the pretreatment measurements, then the outcome of the pre-/posttreatment analysis for the WT serum group would have likely been the same as for the TG serum group, for which there was no difference observed. To see no effect from incubating with serum alone would imply that that an exposure longer than 24 h may be required.

The permeability of the cultures to fluorescein-conjugated 10 kDa dextran was significantly decreased only by treatment with TG serum alone or TG serum in the presence of CSE. Notably, these differences were found only as compared to cells treated with WT serum and not versus cultured incubated in medium alone. In this context, WT serum appeared to decrease permeability, but to a point where it was significantly less than for the medium control group. In addition, permeability measures following treatment with WT serum either alone or in combination with CSE were similar to controls. These findings suggest that, as observed for the TEER studies, that protective factors may be present in the WT serum. One might also question whether previously described protective effects from nicotine could override the toxicity that might be expected to be induced by factors derived from the burned tobacco but not toxicity induced by HIV proteins, inflammatory mediators, and reactive oxidative species present in the HIV serum. This is not supported by the results of the immunofluorescence staining, which demonstrated that exposure to CSE resulted in the most severe disruption of ZO-1 and claudin-5 staining. CSE-induced decreases in TEER and as well as evidence of increased barrier permeability have been also demonstrated using in vitro BBB models employing induced progenitor stem cell-derived BBB cells and BBB cell lines (Kadry et al. 2021; Prasad et al. 2015).

Our studies also showed changes of TJP expression and localization with exposure to CSE or sera +/- CSE. The changes observed for ZO-1 localization are consistent with what has been previously documented for non-confluent cell cultures (Gottardi et al. 1996). In response to cellular signals and to cellular stress, ZO-1 localizes to the nucleus where it interacts with components of the transcriptional machinery to support cellular activation and proliferation (Balda And Matter 2000; Bauer et al. 2010), and in response to cell injury, including toxicity induced by HIV protein (Zhong et al. 2012). Several studies have shown that the HIV-1 Tat protein can reduce the expression and alter the distribution of ZO-1 and claudin-5 as well as the tight junction protein occludin in brain endothelial cells (Andras et al. 2003; Dalvi et al. 2014; Zou et al. 2019). Similarly, HIV-1 gp120 protein can downregulate claudin-5 and occludin expression and increase their phosphorylation levels in these cells. These effects would result in increased paracellular permeability and could potentially facilitate the entry of HIV-1 and inflammatory mediators into the central nervous system. HIV-1 can also indirectly affect tight junction proteins by inducing systemic inflammation and oxidative stress, which can modulate the signaling pathways and transcription factors that regulate tight junction protein expression and localization.

The BBB is also highly permeable to nicotine, for which the permeability increases with chronic exposure (Cisternino et al. 2013). However, acute exposure—which was used in our studies alongside other components of CSE—may have protective effects (Abbaspour et al. 2024; Hu et al. 2024), although only a small percentage of the nicotine present in inhaled cigarette smoke is retained in CSE (Matta et al. 2007). In a study by Hawkins et al. (2004), nicotine infusion in the brains of Sprague-Dawley rats had no effect on TJP expression. However, immunofluorescence imaging of the vessels showed a loss of ZO-1 at cell junctions and nuclear localization of the protein in about 15% of the vessels examined, findings similar to those we obtained from our studies. Via various mechanisms, CS can also potentially impact BBB function by altering TJP expression, localization, and phosphorylation. For example, tyrosine phosphorylation of occludin can decrease its interaction with ZO-1 and promote its endocytosis and degradation (Kale et al. 2003).

Smoking has been linked to suboptimal adherence to ART, reduced ART efficacy and increased toxicity, and accelerated aging in people living with PLHIV (Rahmanian et al. 2011). Our studies further contribute to the understanding of how smoking affects the blood brain barrier, specifically in the context of HIV-1 infection, and suggest mechanisms that may underlie its effects in exacerbating symptoms of HAND. However, there are several limitations that can be addressed in future studies, including quantitative analysis of fluorescence staining performed on replicate samples and following up on the effects on TEER observed for WT serum. Understanding the effects of smoking on BBB integrity and permeability in the context of HIV-1 infection may help optimize clinical management and outcomes for PLHIV who smoke, as well as aid in the prevention and treatment of HIV-1-associated neurocognitive disorders (HAND). Additionally, in vivo studies involving exposure of HIV-1 transgenic rats to cigarette smoke, followed by isolation of brain endothelial cells for in vitro modeling could provide deeper insights into the complex interplay between HIV-1 infection and BBB disruption. Incorporating more advanced and physiologically relevant in vitro BBB models—such as those incorporating multiple cell types or dynamic flow conditions—would further enhance the translational relevance of these findings. Such approaches may help identify novel therapeutic targets to mitigate or prevent neurological complications in individuals living with HIV-1.

## Methods

### Cell cultures

Sprague-Dawley rat brain microvascular endothelial cells (RBMVEC), purchased from Cell Application Inc., were seeded on cell culture flasks coated with Attachment Factor Solution (123 − 100). RBMVEC were maintained at 37 ℃ with 5% CO_2_ in Rat Brain Endothelial Growth Medium (R819-500). The cell culture media components consisted of basal medium, fetal bovine serum, endothelial cell growth supplement, and penicillin/streptomycin solution. Media changes were performed every other day until the cells reached confluency, with subculturing carried out when the cells reached 80–90% confluency, according to the company’s protocol. The cells were used for the assays described below at the third passage. Cell growth in the cultures was monitored using phase-contrast microscopy.

### Transwell cell culture

Clear polyester membrane transwell inserts (6.5 mm diameter, 0.4 μm pore size; Millipore Sigma), were seeded in triplicate on the luminal side at a high density with passage three RBMVEC and cultured in rat brain endothelial cell growth media. Prior to seeding, the upper compartment of the transwell inserts were coated with AFS to allow cells to adhere to the membrane surface. Phase contrast microscopy and transendothelial electrical resistance (TEER) were employed to confirm cell layer confluency and integrity.

### Preparation of Cigarette Smoke Extract (CSE)

Smoke was pulled under vacuum from 3R4F research grade cigarettes (0.7 mg nicotine/cigarettes; Nicotine Research Cigarettes Drug Supply Program, NIDA/NIH) connected to rubber tubing and bubbled through an impinger into 1X phosphate buffer saline (PBS). The CSE solution was then sterile filtered using a 0.22 μm syringe filter (Millipore Sigma) and then stored at -80 °C until use.

### Rat serum

Blood samples were collected from WT Fisher 344 (F344) rats (purchased from Envigo) and HIV-1 transgenic F344 rats (obtained from the Institute for Human Virology, University of Maryland, Baltimore) under ketamine anesthesia via the tail vein. The samples were then centrifuged for 5 min at 1,200 x g at 37 ℃ and then placed at 4 ℃ for 2 h to fix the fibrin clot. The fibrin clot was then squeezed for detachment and centrifuged again for 5 min at 1,200 x g at 37 ℃. The serum was transferred to sterile tubes and centrifuged for 5 min at 1,200 x g at 37 ℃ to remove any contaminating red blood cells. The serum was then incubated in a water bath at 56 ℃ for 30 min to inactivate the complement system and then cooled at room temperature. The serum was aliquoted into individual sterile tubes and stored at – 20 ℃ until use. All procedures were approved by the Morehouse School of Medicine Institutional Animal Care and Use Committee (IACUC).

### Treatment of RBMVEC

Confluent RBMVEC transwell were incubated in fresh medium alone at 37 °C in 5% CO_2_ for 24 h before use. The cultures were then incubated for 24 h in either medium alone, 10% WT rat serum, 10% TG rat serum, 0.50 mg/µL of CSE, 10% WT rat serum containing 0.05 mg/ml CSE, or TG rat serum containing 0.5 mg/ml CSE. In studies by Naik et al., a CSE concentration of 0.5 mg/ml (500 µg/ml) was not demonstrated to have adverse effects on endothelial cell culture viability (Naik et al. 2014). 10% wild-type rat serum was used as a treatment control.

### Trans-endothelial Electrical Resistance (TEER) measurements

TEER (Ω cm^2^) measurements were performed at two different time points after treatment (0 h and 24 h). The measurements were taken using an EVOM (Epithelial Volt/Ohm Meter; World Precise Instruments) equipped with STX2 handheld “chopstick” electrodes, with one in the luminal compartment and the other in the basal compartment. Cell free inserts were also evaluated for TEER. The formula is as follows: **TEER**_REPORTED_ = **R**_RBMVEC_ (Ω) X **M**_AREA_ (cm^2^), with **R**_RBMVEC_ (Ω) = **R**_TOTAL_ – **R**_BLANK_. In this formula, **R**_RBMVEC_ = tissue resistance and **M**_AREA_ = area of the membrane insert. TEER (Ω cm^2^) measurements were obtained on the cell monolayers before treatment to ensure the cells had an equal electrical resistance at baseline.

### Immunofluorescence staining of RBMVEC

Control and treated RBMVEC seeded and grown in four-well chamber slides were fixed in 4% paraformaldehyde (PFA). Fixation was followed by three washes of 1X PBS, and incubation with blocking buffer (1X PBS, 0.5% Triton-X, and 5% donkey serum) at room temperature for one hours. The slides were then incubated overnight at 4℃ with either goat anti-claudin-5 (Abcam, cat. No. EPR7583) or goat anti-ZO-1 (Thermofisher; cat. no. ZO1-1A12) antibodies at a 1:200 dilution. This was followed by incubation with 1:500 dilutions of anti-goat Alexa Fluor 488- or Alexa Fluor 568-conjugated secondary antibodies for two hours at 4℃. The slides were then washed and mounted in DAPI mounting media (Vector) and then imaged using a Zeiss LSM700 confocal laser scanning microscope.

### RBMVEC monolayer permeability measurements

Fluorescein isothiocyanate dextran (FITC-dextran ~ 10 kDa; Sigma) in PBS was added to the luminal compartment of the transwells upon treatment of the cells for 24 h. Transwell inserts containing the FITC-dextran in cell culture media were incubated at 37 ℃ with 5% CO_2_ for 30 min. The media was sampled from the basal compartment and replaced with equal volumes of cell growth media to maintain the same conditions. The the fluorescence intensity of each sample volume was determined at an excitation wavelength of 490 nm and an emission wavelength of 520 nm using a SpectraMax ID5 Microplate Reader (Molecular Devices).Media samples without the FITC-dextran and from the basal compartments of cell-free inserts with FITC-dextran added to the luminal compartment served as references. The permeability measurements were reported as percentages of controls using the following formula: (control – treated)/control x 100.

### Statistical analyses

Mean pretreatment TEER levels were analyzed by one-way analysis of variance (ANOVA) with post-hoc testing using Tukey’s test. Mean pretreatment versus posttreatment TEER values were compared using two-way ANOVA with post-hoc testing using the Šidák correction. For the percent permeability measures, the treatment and control groups were analyzed using one-way ANOVA with post-hoc testing using Tukey’s test. The analyses were performed using GraphPad Prism software. For all analyses a p-value of < 0.05 was considered statistically significant.

## Data Availability

No datasets were generated or analysed during the current study.
